# Calorie restriction and pravastatin administration during pregnancy in obese rhesus macaques modulates maternal and infant metabolism and infant brain and behavioral development

**DOI:** 10.3389/fnut.2023.1146804

**Published:** 2023-05-15

**Authors:** Yu Hasegawa, Danielle H. J. Kim, Zhichao Zhang, Ameer Y. Taha, John P. Capitanio, Casey E. Hogrefe, Melissa D. Bauman, Mari S. Golub, Judy Van de Water, Catherine A. VandeVoort, Cheryl K. Walker, Carolyn M. Slupsky

**Affiliations:** ^1^Department of Food Science and Technology, University of California-Davis, Davis, CA, United States; ^2^Department of Internal Medicine, Division of Rheumatology, Allergy, and Clinical Immunology, University of California-Davis, Davis, CA, United States; ^3^California National Primate Research Center, University of California-Davis, Davis, CA, United States; ^4^The UC Davis MIND Institute, University of California-Davis, Sacramento, CA, United States; ^5^Department of Psychiatry and Behavioral Sciences, University of California-Davis, Sacramento, CA, United States; ^6^Department of Internal Medicine, University of California-Davis, Sacramento, CA, United States; ^7^Department of Obstetrics and Gynecology, University of California-Davis, Davis, CA, United States; ^8^Department of Nutrition, University of California-Davis, Davis, CA, United States

**Keywords:** obesity, pregnancy, gestational weight gain, calorie restriction, pravastatin, infant development, metabolomics, behavior

## Abstract

**Background:**

Maternal obesity has been associated with a higher risk of pregnancy-related complications in mothers and offspring; however, effective interventions have not yet been developed. We tested two interventions, calorie restriction and pravastatin administration, during pregnancy in a rhesus macaque model with the hypothesis that these interventions would normalize metabolic dysregulation in pregnant mothers leading to an improvement in infant metabolic and cognitive/social development.

**Methods:**

A total of 19 obese mothers were assigned to either one of the two intervention groups (*n* = 5 for calorie restriction; *n* = 7 for pravastatin) or an obese control group (*n* = 7) with no intervention, and maternal gestational samples and postnatal infant samples were compared with lean control mothers (*n* = 6) using metabolomics methods.

**Results:**

Gestational calorie restriction normalized one-carbon metabolism dysregulation in obese mothers, but altered energy metabolism in her offspring. Although administration of pravastatin during pregnancy tended to normalize blood cholesterol in the mothers, it potentially impacted the gut microbiome and kidney function of their offspring. In the offspring, both calorie restriction and pravastatin administration during pregnancy tended to normalize the activity of AMPK in the brain at 6 months, and while results of the Visual Paired-Comparison test, which measures infant recognition memory, was not significantly impacted by either of the interventions, gestational pravastatin administration, but not calorie restriction, tended to normalize anxiety assessed by the Human Intruder test.

**Conclusions:**

Although the two interventions tested in a non-human primate model led to some improvements in metabolism and/or infant brain development, negative impacts were also found in both mothers and infants. Our study emphasizes the importance of assessing gestational interventions for maternal obesity on both maternal and offspring long-term outcomes.

## 1. Introduction

Maternal obesity is associated with an elevated risk for pregnancy-related complications. Offspring of mothers with obesity have an increased risk for development of obesity, diabetes, and metabolic syndrome later in life (1), as well as cognitive impairment including autism spectrum disorder (2). However, how maternal obesity leads to increased risk of metabolic disease, as well as cognitive and behavioral impairment in the offspring, is still not completely understood. Some studies have reported on the efficacy of dietary interventions (e.g., calorie restriction and low fat/carbohydrate diet) to control gestational weight gain (GWG) and reduce complications associated with pregnancies in women with obesity with no overt harmful health impacts (3). However, there are large differences in the effectiveness of these interventions due to variances in experimental designs and subject adherence (3–6).

Pregnancy complications that women with obesity experience include development of gestational hypertension and preeclampsia, which if not controlled can progress to eclampsia (1, 4). Pravastatin (which is a statin drug) has recently received attention as a way to treat gestational hypertension and preeclampsia (7, 8). It was previously shown that placentas of women with preeclampsia release excess levels of soluble fms-like tyrosine kinase-1 (sFlt-1), which antagonizes the activity of vascular endothelial growth factor and placental growth factor, leading to endothelial dysfunction (9). In this case, treatment with pravastatin was shown to reduce the secretion of sFlt-1 and attenuate the levels of endothelial dysfunction markers (7). Statins work by inhibiting 3-hydroxy-3-methyl-glutaryl-coenzyme A (HMG-CoA) reductase to lower circulating cholesterol levels. However, pravastatin is a less potent HMG-CoA reductase inhibitor than other statins, and it is specific to hepatocytes (8). Statins have been reported to attenuate inflammatory markers [e.g., high-sensitivity C-reactive protein (hs-CRP), interferon-γ (IFN-γ), tumor necrosis factor-α (TNF-α)], as well as improve imbalance in the T helper cell type 1 (Th1) over Th2 cytokine responses observed in preeclampsia (8). There are mixed results regarding whether pravastatin can cross the placenta. For example, by using the technique of dual perfusion of the placental lobule, Nanovskaya *et al* found that the transplacental transfer and distribution of pravastatin was rapid and bidirectional (10), whereas Zarek *et al* found it slow and limited (11). Although statin use during pregnancy has been accompanied with a concern of inducing fetal malformation and aberrant brain development (12), no detectable safety issues have been reported (7, 8, 13).

To date, most intervention studies have focused on short-term overt pregnancy outcomes for both mothers (e.g., GWG, gestational diabetes mellitus, preeclampsia) and infants (e.g., perinatal death, still birth, macrosomia) (3, 7, 8), but little has been done to evaluate the metabolic and cognitive/affective development of offspring. Therefore, in this study, we tested two gestational interventions (gestational calorie restriction or pravastatin administration) with the hypothesis that they could normalize metabolic dysregulation in pregnant mothers and infants, which would improve infant cognitive/social development. To test our hypothesis, a non-human primate model was utilized, and ^1^H nuclear magnetic resonance (NMR)-based metabolomics was employed to assess the impact of two different obesity interventions on maternal and infant metabolism. We also considered potential impacts on the offspring brain by assessing mechanistic target of rapamycin complex 1 (mTORC1) activity through measurement of Akt, AMPK, and p70-S6K, as well as behavioral analyses. We assessed mTOR activity in the brain as it is critical for neurodevelopment (14, 15) because it plays important regulatory roles in neuronal and glial differentiation, neural stem cell function (16), and synaptic protein synthesis (14). Our study deepens the understanding of the impacts of calorie restriction or pravastatin administration in mothers with obesity on maternal and offspring health.

## 2. Materials and methods

### 2.1. Study population

Pregnant female rhesus macaques (*Macaca mulatta*) from an indoor breeding colony at the California National Primate Research Center with appropriate social behavior and previous successful pregnancies were enrolled. Animal handling was approved by the UC Davis Institutional Animal Care and Use Committee (IACUC) (#19299). A qualitative real-time PCR assay (17) was used to identify mothers with male fetuses to include in this study. Since obesity is defined as subjects with body fat above 30% for women, according to guidelines from the American Society of Bariatric Physicians, American Medical Association, and in some publications (18, 19), a Body Condition Score (BCS) of 3.5 [32.8% body fat on average (20)] was used as the cutoff. Therefore, mothers with BCS of 3.5 and above were categorized as obese. Obese mothers were randomly assigned to the Obese Control (OC) group, OR group (received calorie Restriction), or OP group (received Pravastatin). Mothers with BCS of 2.5 and below were assigned to the Lean Control (LC) group. A summary of the animals used in this study is in Supplementary Table S1. The unbalanced sample size (Supplementary Table S2) was because some mothers were removed from the analyses due to fetal deaths for unknown reasons, misidentification of a female fetus, different timing for study enrollment, or technical issues upon collecting samples. The number of animals was six for the LC, seven for the OC, five for the OR, and seven for the OP groups.

### 2.2. Feeding, rearing, and interventions

Adult female animals were provided monkey diet (High Protein Primate Diet Jumbo #5047; LabDiet, St. Louis, MO, USA) twice a day between 6–9 am and 1–3 pm. The calories were provided as 56% from carbohydrates, 30% from protein, and 13% from fat (more detail is available in Supplementary Table S1). Mothers in the LC, OC, and OP groups were fed nine biscuits twice a day once pregnancy was confirmed. Mothers in the OR group received a restricted supply of food once the pregnancy was detected and was maintained throughout pregnancy. The food restriction was set such that the average total weight increase would be 8% body weight from the last day before conception because the recommended total weight gain in the 2nd and 3rd trimesters is 5–9 kg for the average US woman with obesity who weighs 80 kg and is 1.6 m in height (Body Mass Index of 30), according to the Institute of Medicine 2009 guidelines (21). The actual number of biscuits provided to each mother is summarized in Supplementary Table S2. During nursing of infants older than 4 months, all mothers were provided 12 biscuits. Fresh produce was provided biweekly, and water was provided *ad libitum* for all mothers. Mothers in the OP group were given pravastatin sodium (ApexBio Technology, Houston, TX, USA) at 20 mg/kg body weight prepared in a neutralized syrup [20 mg/mL sodium bicarbonate dissolved in a fruit-flavored syrup (Torani, San Leandro, CA, USA)] once a day from the time pregnancy was confirmed until delivery. The caloric value of the administration was made so as not to influence body weight or skew nutritional value of the diet among all treatment groups. Both interventions were applied only during gestation. Although most mothers were allowed to deliver naturally, cesarean delivery was performed for fetal indications when recommended by veterinarians (2 for each of the LC and OC groups, and 1 for the OP group; Supplementary Table S3). These mothers did not accept her infant following birth, so foster mothers were provided (Supplementary Table S3).

### 2.3. Sample collection and pre-processing prior to sample storage

The animal caretakers and researchers who collected samples were blinded for group assignment by coding all animals by IDs. The collected biological samples were randomized by using random numbers and the group assignment was blinded during the data collection.

Both mothers (during pregnancy) and infants were weighed every week. One day before sample collection, food was removed 30 min after the afternoon feeding, and biological samples were collected prior to the morning feeding. To collect biological samples, animals were anesthetized using 5–30 mg/kg ketamine or 5–8 mg/kg telazol. Both maternal and infant blood was collected using 5 mL lavender top (EDTA) tubes (Monoject, Cardinal Health, Dublin, OH, USA) and urine was collected from the bladder by ultrasound-guided transabdominal cystotomy using a 22-gauge needle and stored in a 15 mL Falcon tube. A placental sample was collected at GD150 transabdominally under ultrasound guidance using an 18-gauge needle attached to a sterile syringe. Sample processing was as previously described in (22). Necropsy was conducted between 9:30 am-1:30 pm. First, infants at the age of PD180 were fasted and anesthetized with ketamine, and plasma and urine were collected. Then, euthanasia was performed with 120 mg/kg pentobarbital, followed by heparin injection, clamping of the descending aorta, and flushing with saline until clear. The kidney and brain (amygdala, hippocampus, hypothalamus, and prefrontal cortex) were collected, weighed, and immediately frozen on dry ice or liquid nitrogen and stored at −80°C until further analyses.

### 2.4. Metabolite extraction and analysis by ^1^H NMR, and measurement of insulin, cholesterol, cytokine, and cortisol

Detailed procedures were previously described (22). Briefly, plasma and urine samples were filtered using Amicon Ultra Centrifugal Filter (3 k molecular weight cutoff; Millipore, Billerica, MA, USA), and the supernatant was used for analysis. For both the placental and brain tissue samples, polar metabolites were extracted using our previously reported method (23). A total of 180 μL of sample (tissue extract or filtered urine or serum) was transferred to 3 mm Bruker NMR tubes (Bruker, Billerica, MA, USA). Within 24 h of sample preparation, all ^1^H NMR spectra were acquired using the noesypr1d pulse sequence on a Bruker Avance 600 MHz NMR spectrometer (Bruker, Billerica, MA, USA) (24). Chenomx NMRSuite (version 8.1, Chenomx Inc., Edmonton, Canada) (25) was used to identify and quantify metabolites.

Heparin-treated plasma samples were used to measure insulin and 17 cytokines and chemokines (hs-CRP, Granulocyte-macrophage colony-stimulating factor, IFN-γ, TNF-α, transforming growth factor-α, monocyte chemoattractant protein-1, macrophage inflammatory protein-1β (MIP-1β), and interleukin (IL)-1β, IL-1 receptor antagonist (IL-1ra), IL-2, IL-6, IL-8, IL-10, IL-12/23 p40, IL-13, IL-15, and IL-17A) using a multiplex Bead-Based Kit (Millipore) on a Bio-Plex 100 (Bio-rad, Hercules, CA) following the manufacturer’s protocol. For each sample, a minimum of fifty beads per region were collected and analyzed with Bio-Plex Manager software using a 5-point standard curve with immune marker quantities extrapolated based on the standard curve. Two samples were removed for analysis of TNF-α and IL-1ra as technical errors (both from Animal ID 1132103: 895.2 and 1115.1 pg./mL at gestational days (GD) 90; 510.8 and 617.2 pg./mL at GD120, respectively). Plasma cholesterol level was measured by Clinical Laboratory Diagnostic Product (OSR6116) on Beckman Coulter AU480 (Beckman Coulter, Brea, CA).

Infant plasma cortisol level at PD110 was assessed as previously described (26, 27). In short, infants were transferred to a test room at 9 am and blood was drawn at 11 am (Sample 1), followed by another blood collection at 4 pm (Sample 2) and intramuscular injection of 500 μg/kg dexamethasone (Dex) (American Regent Laboratories, Inc., Shirley, NY). On the next day, a blood sample was collected at 8:30 am (Sample 3), and then 2.5 IU of adrenocorticotropic hormone (Amphastar Pharmaceuticals, Inc. Rancho Cucamongo, CA) was injected intramuscularly. The last blood was collected (Sample 4) 30 min after adrenocorticotropic hormone injection. The collected blood samples were processed and stored, and cortisol concentration was assessed by a chemiluminescent assay on the ADVIA Centaur CP platform (Siemens Healthcare Diagnostics, Tarrytown, NY, USA).

### 2.5. Protein quantification and western blot analysis

Protein quantification and western blot analysis were conducted as previously described (23). Briefly, a DC Protein Assay kit (Bio-Rad) was used for total protein quantification. For the western blot, the following antibodies were used: rabbit anti-Akt (#9272), anti-phospho-Akt (#9275; Thr308), anti-Adenosine Monophosphate-activated Protein Kinase (AMPK)α (#2603), anti-phospho-AMPKα (#2535; Thr172), anti-p70 S6 Protein Kinase (S6K) (#9202), anti-phospho-p70 S6K (#9234; Thr389), and goat anti-rabbit IgG antibody conjugated to horseradish peroxidase (#7074) (Cell Signaling Technology, Danvers, MA, USA). For chemiluminescent detection, Clarity Western ECL Blotting Substrates (Bio-Rad) or Radiance Plus (Azure biosystems, Dublin, CA USA) were used.

### 2.6. Visual paired comparison test at ~1 month of age

On post-conception day 200 ± 3 days, recognition memory development of infants was assessed using Visual Paired Comparison (VPC) conducted between 8:30–10:30 am (28–30). In brief, two identical black and white contrast abstract pictures (Fagan Test of Infant Intelligence; Infantest Corporation, Cleveland, OH) were placed right and left of center, and infants were allowed to observe for a total of 20 s (familiarization trial). Then, the pictures were switched to the familiar and novel ones placed either right or left of center based on a predetermined random order, and the frequency and duration of looking were video recorded using The Observer software (Noldus, Inc., Wageningen, The Netherlands) for a 10 s from the time of the first fixation (preference trial 1). The side of the pictures was switched, and another 10 s test period was recorded (preference trial 2). In total, four problems were conducted to each infant. Novelty preference was calculated by dividing the number of fixations at the novel stimulus by number of fixations at both the novel and familiar stimulus.

### 2.7. Human intruder test at ~4 months of age

Human intruder (HI) test was conducted as described before (27). Briefly, we measured the frequency of scratch [as an indicator of anxiety (31)] for 1 min in four graded levels of stress: Profile-Far (the left profile of a technician was presented from ~1 m away from an infant), Profile-Near (left profile presented from ~0.3 m), Stare-Far (direct eye contact was made with the infant from far position), and Stare-Near (direct eye contact made from near position).

### 2.8. Statistical analyses

All statistical analyses were done in R (version 3.6.1). GWG was obtained using the following equation: [(delivery weight) - (last weight before conception)]/(last weight before conception) * 100, where the delivery weight is the last weekly weight before delivery (Supplementary Table S3). Homeostasis model assessment for insulin resistance (HOMA-IR) was obtained using the following equation: [fasting glucose (mg/dL) * fasting insulin (μU/mL)]/405. One animal with ID1215218 (OC group) was removed due to a technical error in measurement of insulin, as the insulin level was higher than biologically reasonable (32). The following equation was used to calculate estimated placental volume (EPV): (πT/6)*[4H (W − T) + W (W − 4 T) + 4 T^2^], where T is thickness at maximal height, H is height at maximal width, and W is maximal width measured by ultrasound (33).

Either the OR or the OP group was separately compared with both control groups (OC and LC). For data that were collected at a single time point, one-way ANOVA was performed and Tukey honestly significant difference as a post-hoc test was applied. Eta squared (η^2^) was calculated as the effect size measurement using sjstats package (version 0.18.0), with a medium effect defined as 0.06<η^2^ < 0.14, and a large effect defined as η^2^ ≥ 0.14. For data collected at multiple time points, a linear mixed-effects model was fit followed by ANOVA to test for group differences using the lme4 package (version 1.1.21). Estimated marginal means was calculated by using emmeans (version 1.4.4) and pairwise comparison was applied as a post-hoc test. Effect size for multiple time points was calculated as coefficient of determination (*R*^2^) using r2glmm (version 0.1.2), with a medium effect defined as 0.15 < *R*^2^ < 0.35, and a large effect defined *R*^2^ ≥ 0.35.

For metabolomics data, metabolite concentrations were log-transformed prior to statistical analysis. Benjamini-Hochberg false discovery rate for multiple testing correction was applied. After testing for the model fit, the statistical model was adjusted by maternal age for maternal plasma samples, by the centered GWG values for maternal urine samples, and by maternal age for infant plasma samples. Infant weights were corrected for gestational length. In order to avoid false negative results due to the small sample size, statistical significance was defined as a combination of uncorrected *p*-value < 0.1 and medium-large effect (34). Unless otherwise stated, data are presented as mean ± standard error. We used duration of looking from eye tracking test measured at months 0.25, 1, 3, and 6 as the primary outcome to calculate the power. Based on a previous study (35), we assumed a correlation among the four repeated observations ranged between 0.3 and 0.7. For a type one error where α = 0.05, the intended sample size of eight monkeys per group can provide a power of 80–93% to detect a rate of change per month between the two groups as small as 1.1 SD (36).

For cytokine/chemokine data, Partial Least Squares Discriminant Analysis (PLS-DA) was performed to examine whether different combinations of multiple cytokines could be used to differentiate between treatment groups. The PLS-DA model was used to cluster the groups and the Variable Importance in Projection (VIP) scores were obtained as a measure of the importance of specific cytokines/chemokines in the PLS-DA model. Cytokines/chemokines with VIP scores above 1.0 were defined to have meaningful impact on group separation. PLS-DA was performed using MetaboAnalyst 5.0 (37).

## 3. Results

### 3.1. Characteristics of animals

Group assignment was based on pre-gestational maternal adiposity assessed using BCS, which ranges from 1 to 5 (38). Pregnant female rhesus macaques with a BCS less than 2.5 (estimated average body fat 19.7%) were prospectively assigned to the lean control (LC) group, while those with a BCS above 3.5 [estimated average body fat 32.8% (20)] were assigned to either the obese control (OC) group or two groups that received interventions during pregnancy: calorie restriction (OR) or daily administration of pravastatin from confirmed conception until delivery (OP) (Table 1; Supplementary Table S3). Obese animals were generally older than lean ones, but OR and OP mothers were significantly older than LC mothers. Pre-pregnancy weights of monkeys in the OC, OR and OP groups did not differ, but were significantly higher than monkeys in the LC group. Throughout the study, statistical significance was defined by a combination of *p*-value < 0.1 and medium-large effect size in order to account for the relatively small sample size and the inherent variability in the rhesus macaque dataset. Mean GWG rate was significantly higher in the LC group compared to the three obese groups (*p* < 0.001 when comparing LC vs. OC, LC vs. OR, or LC vs. OP). Although a 39.2% calorie reduction was applied on average to OR mothers as a dietary restriction, all three obese groups showed a mean GWG rate of less than 8%, with no significant difference in the intervention groups (Table 1).

**Table 1:** Summary of the BCS, age, pre-pregnancy weight, and GWG rate.

	LC	OC	OR	OP
BCS	2.2 ± 0.4	3.8 ± 0.3	3.5 ± 0	3.7 ± 0.4
Age (years)	8.9 ± 1.3^a^	10.2 ± 1.4^ab^	11.7 ± 2.1^b^	11.6 ± 1.5^b^
Pre-pregnancy weight (kg)	6.9 ± 1.1^a^	10.8 ± 1.2^b^	10.0 ± 1.4^b^	10.0 ± 0.9^b^
GWG rate (%)	34.6 ± 17.4^a^	7.4 ± 5.1^b^	4.4 ± 2.5^b^	4.7 ± 8.4^b^

Data are expressed as mean ± standard deviation. Age, pre-pregnant weight and GWG rate were compared between the groups (LC and OC groups were compared with OR or OP groups) by one-way ANOVA test followed by Tukey’s post-hoc test. Different superscript letters indicate a significant group difference by the post-hoc test. This table includes mothers with successful delivery only. BCS, body condition scores; GWG, gestational weight gain; LC, Lean Control; OC, Obese Control; OR, Obese mothers with gestational calorie Restriction; OP, Obese mothers with gestational Pravastatin administration.

### 3.2. The impact of the interventions on metabolism and immunity

**Figure 1:**
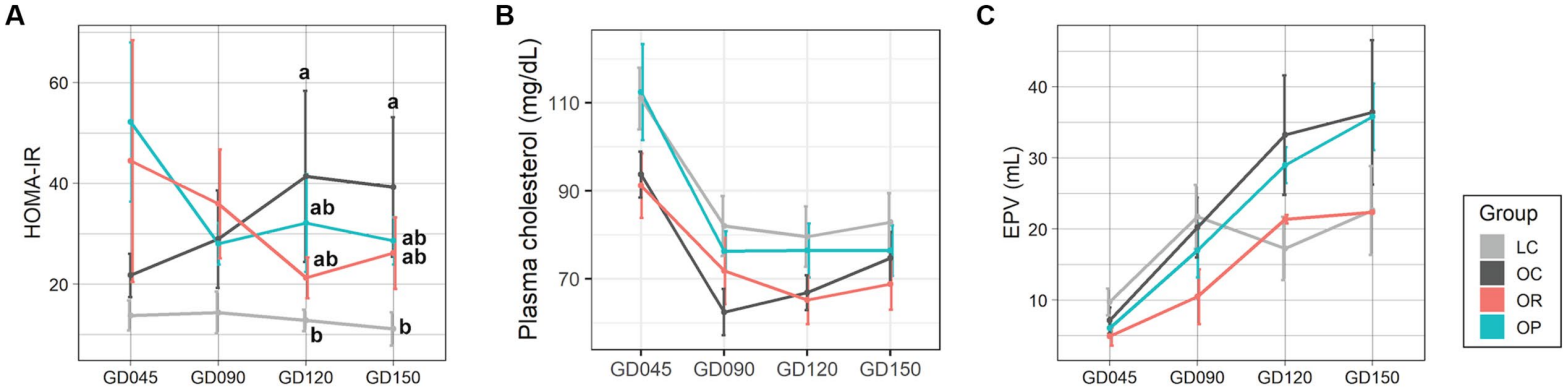
Assessments of the maternal metabolism during pregnancy. Line plots showing the levels of **(A)** HOMA-IR, **(B)** plasma total cholesterol, and **(C)** EPV. Data are presented as mean ± standard error as error bars. The gray, black, red, and blue colors correspond to the LC, OC, OR, and OP groups, respectively. Letters represent the statistical significance by post-hoc test. EPV, estimated placental volume; GD, gestational day; HOMA-IR, homeostasis model assessment for insulin resistance.

Maternal plasma and urine samples were collected once during the 1st and 2nd trimesters (at GD45 and 90) and twice during the 3rd trimester (GD120 and GD150), and a placental sample was collected at GD150 (Supplementary Tables S3, S4). Concentrations and results of statistical analyses of plasma, urine, and placental metabolites are summarized in Supplementary Tables S5–S7, respectively. Dietary restriction applied to OR mothers did not result in hypoglycemia as the level of fasting blood glucose was within the normal range (39) throughout the pregnancy (Supplementary Table S5). While OC mothers showed a progressive and significant increase in insulin resistance measured by HOMA-IR over time, both OR and OP mothers showed decreasing trends, approaching levels found in LC mothers with no statistically significant differences between groups (Figure 1A). Plasma cholesterol in the OP group was similar to the LC group, which was generally higher than in the OC and OR groups (Figure 1B). Furthermore, the impact of maternal obesity and interventions on the size of the placenta was also assessed by EPV (Figure 1C). The EPV of the OR group was lower than the control groups during the earlier part of pregnancy but was similar in size as the LC group in the later part of pregnancy. Specifically, the EPV of the OR group was lower than that of the OC mothers by 32, 48, 36, and 39% at each time point, respectively. The EPV of the OP group was not significantly different from the OC group throughout pregnancy.

**Figure 2:**
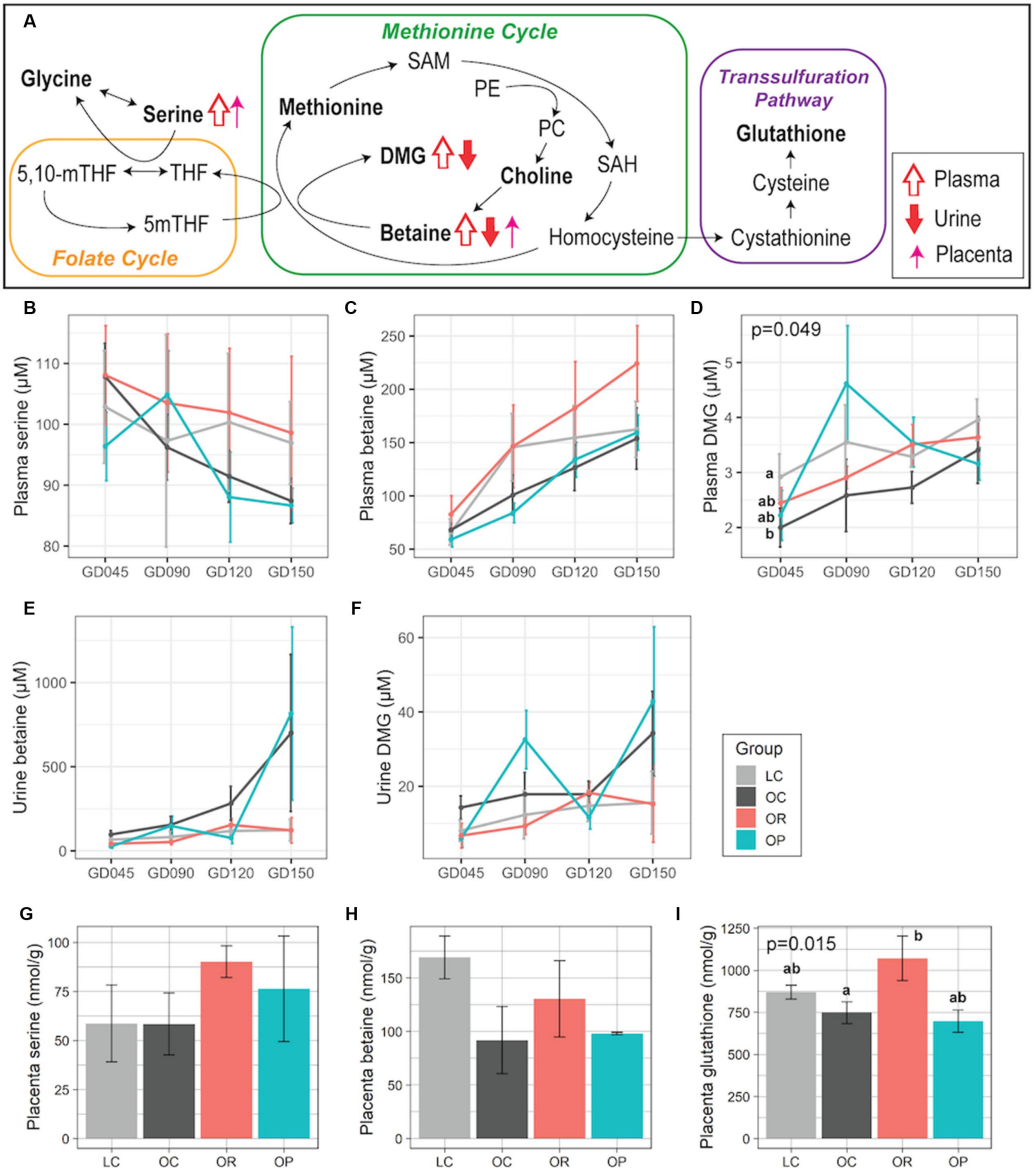
Maternal plasma, urinary, and placental metabolites involved in one-carbon metabolism. **(A)** Schematic summary of the trends found in OR mothers compared to OC mothers. Trends in plasma, urine, and placental samples are expressed with open, closed, and thin arrows, respectively. Metabolites profiled in this project are in bold. Line plots showing concentrations of plasma **(B)** serine, **(C)** betaine, **(D)** DMG; urinary **(E)** betaine, and **(F)** DMG. Bar plots showing placental **(G)** serine, **(H)** betaine, and **(I)** glutathione. For plasma and urine samples, a linear mixed-effects model was fitted followed by ANOVA to test for group differences. *p*-values comparing the LC, OC, and OR groups were noted if significant. For placental samples, eta-squared was used as effect size measurement. Data are presented as mean ± standard error as error bars. Letters represent the statistical significance by post-hoc test applied to compare the LC, OC, and OR groups. The gray, black, red, and blue colors correspond to the LC, OC, OR, and OP groups, respectively. DMG, N,N-dimethylglycine; GD, gestational day; PE, phosphatidylethanolamine; PC, Phosphatidylcholine; SAH, S-adenosylhomocysteine; SAM, S-adenosylmethionine.

We were particularly interested in the one-carbon metabolism pathway because our previous study found that OC mothers had alterations in the levels of metabolites in this pathway compared to LC mothers (22), and others have also found an association between perturbations in this pathway and adverse neurodevelopmental outcomes (40, 41). The impact of calorie restriction in mothers with obesity was apparent (Figure 2A). While plasma serine showed a decreasing trend over pregnancy in all groups, the mean level was higher in OR mothers compared with OC mothers, and more similar to LC mothers (Figure 2B). Plasma betaine and N,N-dimethylglycine (DMG) were higher in OR mothers compared to OC mothers (Figures 2C,D), and more similar to LC mothers. Similarly, urinary betaine and DMG were similar between the OR and the LC groups (Figures 2E,F). In the placenta, OR mothers showed higher levels of serine, betaine, and glutathione (*p* = 0.015 and eta-squared (η^2^) = 0.81) compared to the OC mothers, but was not significantly different from the LC mothers (Figures 2G–I). The metabolome of OP mothers was not significantly different from OC mothers with respect to metabolites in the one-carbon metabolism pathway, although levels of plasma and urinary DMG tended to fluctuate throughout pregnancy (Figures 2D,F).

There were no statistically significant group differences for the maternal cytokine/chemokine levels. However, our VIP analysis showed several cytokine/chemokines that were driving the trending immune differences between OR/OP and OC longitudinally (Supplementary Figure S1). The OR group displayed a trend toward having lower levels of inflammatory-related cytokines/chemokines such as IL-1β, MIP-1α, MIP-1β, and IFN-γ, but higher levels of anti-inflammatory IL-10 that distinguished them from the OC group according to the PLS-DA VIP scores. The pattern was similarly observed when comparing the OP and the OC groups. The OP group had lower levels of pro-inflammatory IL-1β and higher levels of anti-inflammatory cytokines (e.g., IL-ra, IL-13) and during late pregnancy.

### 3.3. The impact of gestational interventions on offspring metabolism

**Figure 3:**
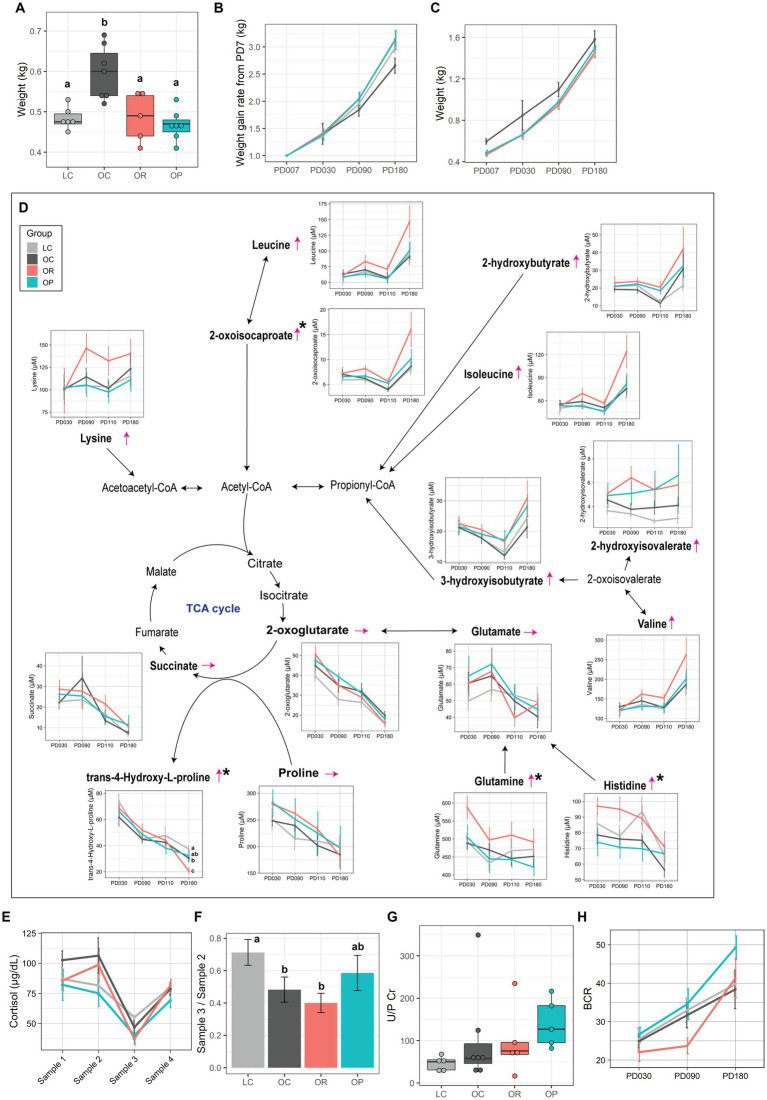
The impact of interventions on offspring metabolism. **(A)** Boxplot showing early infant weight measured at PD7. Line plots showing **(B)** weight gain rate from infant weight at PD7 and **(C)** infant weight. **(D)** Schematic summary of the infant plasma metabolites involved in energy metabolism. Trends found in the plasma metabolites of OR infants compared to that of OC infants are expressed with thin red arrows. The black asterisk represents metabolites that showed significant group difference between LC, OC, and OR groups (*p* < 0.05 before FDR correction). Letters represent the statistical significance by post-hoc test. Metabolites profiled in this project are in bold. **(E)** Line plot showing the level of plasma cortisol over 4 sampling time points (post-hoc test found no significance between groups at any time points). **(F)** Bar plot representing suppression in plasma cortisol level made by Dex (Sample 3/Sample 2). Assessment of infant kidney function by the **(G)** U/P Cr, and **(H)** BCR. Data are presented as mean ± standard error as error bars. The gray, black, red, and blue colors correspond to the LC, OC, OR, and OP groups, respectively. BCR, blood urea nitrogen over plasma creatinine levels; CoA, coenzyme A; U/P Cr, ratio of urinary over plasma creatinine levels; PD, postnatal day; TCA cycle, tricarboxylic acid cycle.

Infant weight was measured on postnatal day (PD) 7. OR and OP infants showed significantly lower early weights than OC infants at PD7 (*p* < 0.01), which was within the typical birthweight of rhesus macaques [0.4–0.55 kg (42)], and as low as LC infants (Figure 3A). Although weight gain rate from PD7 did not differ among groups prior to weaning (around PD30), even with a correction for gestation length, during the weaning period both OR and OP infants had the highest rate of weight gain, followed by LC, and then OC infants with no significant difference (Figure 3B). Despite the lower weight gain rate found in OC infants, their weights remained higher than infants of other groups throughout the experiment (Figure 3C).

Metabolomics analysis of plasma samples collected at PD30, 90, 110 and 180, as well as urine and brain samples collected at necropsy on PD180, are summarized in Supplementary Tables S8–S13. OR infants showed higher levels of the following plasma metabolites in comparison to LC and/or OC infants: 2-oxoisocaproate (*p* = 0.09 and *R*^2^ = 0.26), glutamine (*p* = 0.08 and *R*^2^ = 0.26), and histidine (*p* = 0.01 and *R*^2^ = 0.39) although post-hoc tests did not find significance, and trans-4-Hydroxy-L-proline (*p* = 0.04 and *R*^2^ = 0.31) at PD180 (Figure 3D). Although a significant group difference was not found after adjusting for maternal age, TCA cycle-related metabolites, lysine, leucine, 2-hydroxybutyrate, isoleucine, and valine, were higher in concentration at PD180. No metabolites were significantly different in mothers provided gestational pravastatin.

The levels of cytokine and chemokines in infant plasma samples were assessed at PD30, 90, and 180. While none of the infants displayed any significant group differences, using PLS-DA analysis, we noted that OR infants had lower levels of lipid/glucose metabolism-related metabolic markers (e.g., C-peptide, PP, GIP) than OC infants at PD30, based on their VIP scores (Supplementary Figure S2). This pattern was not observed when comparing OP infants to OC infants at PD30, and no distinct immune/metabolic pattern was observed at PD30. At PD180, OP infants trended toward higher levels of inflammatory chemokines (e.g., MCP-1, MIP-1α) and lower IL-8 than OC infants.

Since we previously found that OC infants showed a phenotype of Dex super-suppressor (22), we assessed the impact of gestational interventions on the cortisol regulation in infants. Plasma samples were collected at PD110 in four different conditions (refer to the Method section). While the level of cortisol did not differ between groups at any time point (Figure 3E), we examined group differences in negative feedback sensitivity by examining the ratio of Sample 3 (Dex-suppressed values obtained at 9:30 am on the second day) to Sample 2 (values obtained at 4 pm on Day 1, immediately preceding the Dex administration). OR infants showed similar ratios as OC infants, which were significantly lower than LC infants (*p* < 0.01 and η^2^ = 0.37; Figure 3F). However, the ratio for OP infants was more similar to the ratio found in LC infants.

Analysis of the infant urinary metabolome revealed that infants of the CR group had only one metabolite, fucose, that was significantly different from the LC group (*p* = 0.030 and η^2^ = 0.38) (Supplementary Table S9). However, infants in the OP group had major differences in their urine metabolome, with 35 metabolites significantly different between groups. Notably, 28 of them are related to microbial activity, with the following metabolites significantly higher in the OP group compared to the OC and LC groups: 3-indoxyl sulfate, 4-hydroxyphenyllactate, allantoin, glutamate, inosine, trimethylamine, and trimethylamine N-oxide (Supplementary Table S8). Lastly, because we observed significant differences in urinary metabolites between infants in the OP group and the OC and LC groups, we assessed markers of infant kidney function: urine over plasma creatinine level (U/P Cr) and blood urea nitrogen (BUN) over plasma creatinine level (BCR) (43, 44). OP infants tended to show higher levels of U/P Cr (Figure 3G) and BCR (Figure 3H) compared to LC, OC, and OR infants.

### 3.4. Impact of the interventions on offspring brain function and cognitive/social development

**Figure 4:**
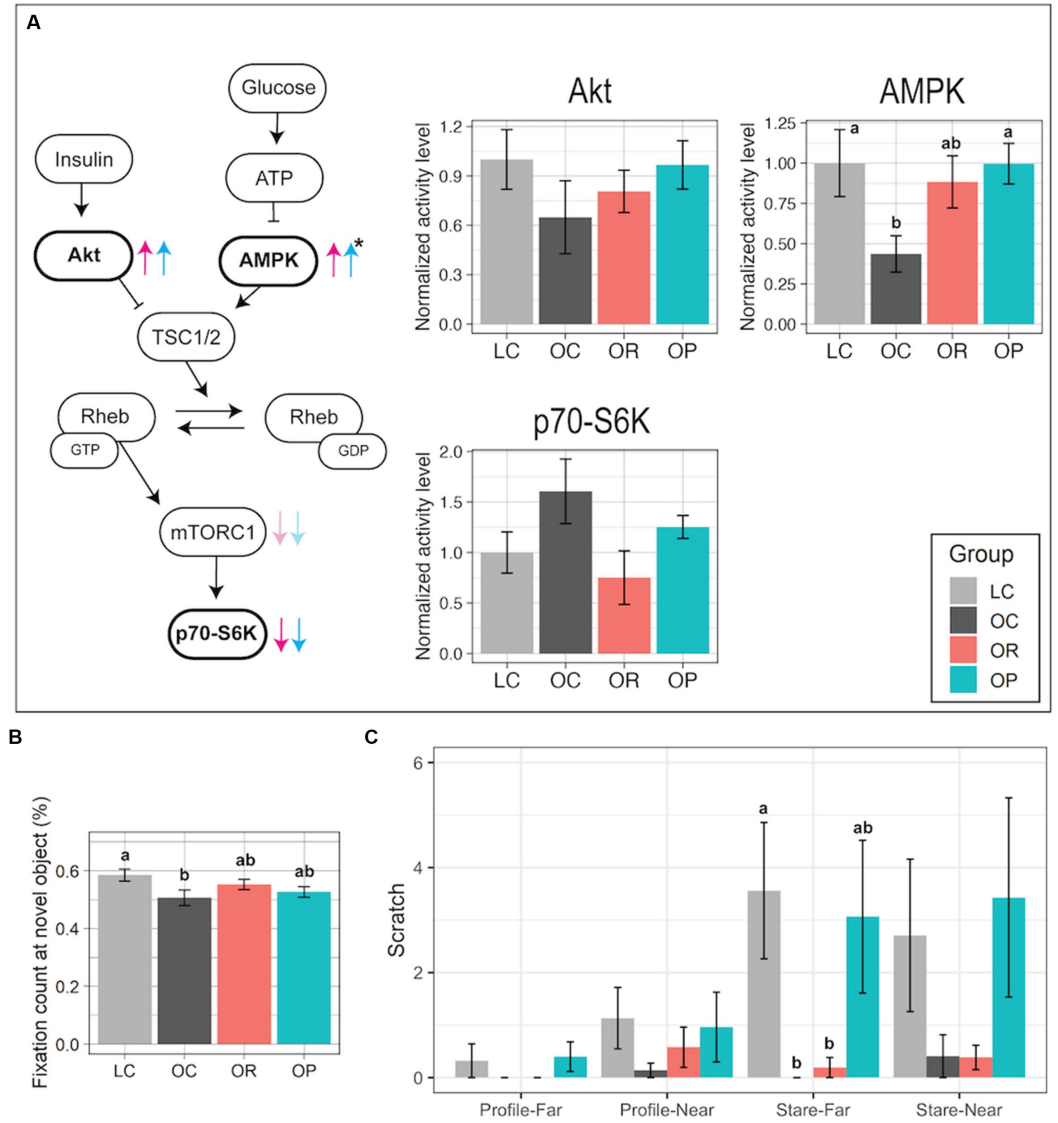
Assessment of the brain mTOR protein pathway activity and cognitive and emotional development. **(A)** Schematic summary of the mTOR signaling pathway with bar plots representing normalized activity levels of Akt, AMPK, and p70-S6K in the prefrontal cortex. In comparison to the activity levels found in the OC group, the trends found in the OR or OP groups are shown as red and blue arrows, respectively. The black asterisk represents a significant group difference by one-way ANOVA. **(B)** Bar plot showing the proportional fixation count at the novel object (VPC test). **(C)** Bar plot showing the frequency of scratch measured in either Profile-Far (a technician presented the profile face from ~1 m away from the infant in a cage), Profile-Near (profile face presented from 0.3 m away), Stare-Far (a technician stared into the eyes of the infant from ~1 m away), or Stare-Near (stared from 0.3 m away) condition (HI test). Data are presented as mean ± standard error as error bars. Letters represent the statistical significance by the post-hoc test. The gray, black, red, and blue colors correspond to the LC, OC, OR, and OP groups, respectively. Rheb, Ras homolog enriched in brain; TSC1/2, Tuberous sclerosis proteins 1 and 2.

The impact of the interventions on the activities of proteins in the brain mTORC1 signaling pathway was assessed because our previous findings suggested that its activity was elevated in the prefrontal cortex of OC infants (22). AMPK activity levels in the prefrontal cortex were significantly different between the control groups and the intervention groups with a large effect size (*p* = 0.050 and η^2^ = 0.33 for OR vs. control groups; *p* = 0.021 and η^2^ = 0.37 for OP vs. control groups; Figure 4A; Supplementary Figures S3, S4). Post-hoc tests determined that AMPK activity trended higher in the OR group compared to the OC group (*p* = 0.073), and in the OP group compared to the OC group (*p* = 0.014), and both were similar to the level found in the LC group. In addition, the intervention groups showed lower p70-S6K activity than in the OC group, suggesting that they had similar mTORC1 activity as the LC group. Lastly, mean Akt activity was slightly higher in the intervention groups than in the OC group, approaching the level found in the LC group. Other brain regions did not show any significant differences among groups (Supplementary Figure S4).

A VPC test was applied on post-conception day 200 ± 3 days (~1 month of age) to assess early brain development. Typically developed animals show preference in observing a novel object over a familiar one, showing higher frequency of looks at a novel object (45, 46). In our study, LC infants had significantly higher novel object fixation count compared to OC infants (Figure 4B). The interventions had a minor impact on improving the novelty preference as the post-hoc test did not find significant group differences in the proportional fixation count at the novel object between the intervention group and the control groups (Figure 4B).

A HI test was utilized to assess anxious responding to threat in infants at PD110. It was composed of four 1 min trials that involved graded challenges, and the frequency of scratch made by each infant was recorded as the measurement of anxiety (31). LC infants showed a higher frequency of scratch in more challenging conditions (Profile-Near than in Profile-Far, as well as in Stare conditions than in Profile conditions; Figure 4C). In contrast, OR and OC infants’ behavior did not differentiate conditions: they showed little difference in frequency of scratch between the four conditions, low frequencies in all conditions, and a significantly lower frequency in Stare-Far compared to LC infants (*p* < 0.01 and η^2^ = 0.49). On the other hand, the results found in OP infants were more similar to those found in LC infants.

## 4. Discussion

Beginning a pregnancy obese may have health risks for both mothers and their offspring (1, 2, 4). Prior work has addressed the incidence of short-term pregnancy complications (4, 7, 8), but long-term assessment is missing. In this study, we applied two interventions during gestation to obese pregnant rhesus macaques, calorie restriction and pravastatin administration. We then evaluated their impacts on both mothers and infants. Although the two interventions tested in this study led to some improvements in maternal metabolism and infant behavior, unique negative impacts were also found both in mothers and their offspring.

For one of the interventions, we applied mild to moderate calorie reduction (47) during pregnancy in order to achieve the recommended GWG by the Institute of Medicine 2009 guidelines (21). As a result, some positive impacts on maternal metabolism were found. First, HOMA-IR showed an improvement over the course of pregnancy, approaching the levels found in LC mothers. Second, calorie restriction normalized concentrations of metabolites involved in one-carbon metabolism to be more similar to those found in LC mothers as observed in the levels of plasma betaine and DMG. Considering that the conversion of betaine to DMG is coupled with the conversion of homocysteine to methionine, which is critical for a healthy pregnancy as the demand for methylation increases in later pregnancy (48), gestational calorie restriction might have normalized the altered methylation profiles found in OC mothers. Third, calorie restriction in obese mothers normalized the EPV and early infant weights to the levels found in LC mothers. These results suggest that placental size and function of the OR group were altered to supply less nutrients to the fetuses compared to the OC group.

In our study, OR infants did not show significant improvement in cognitive performance from that of OC infants, and their affective response was inappropriate as the animals did not differentiate between graded conditions of threat like the OC infants. Another potential negative impact of gestational calorie restriction is that OR infants exhibited a higher rate of weight gain, energy metabolism, and protein metabolism that appeared to favor protein catabolism compared to OC infants. While we cannot rule out the fact that OR mothers in this study were provided the same amount of food as other mothers in the study in the postpartum period, which might have created a mismatched nutritional environment for their offspring: undernourishment *in utero* and over-nutrition after birth, these results suggest that infants born to mothers who restricted calories during pregnancy may experience metabolic perturbation later in life.

The other intervention in this study was administration of pravastatin to obese mothers once a day during pregnancy. Pravastatin inhibits HMG-CoA reductase, and therefore lowers circulating cholesterol (49, 50). In our study, levels of total plasma cholesterol were higher in the LC group than in the OC group throughout pregnancy, which may suggest that the OC group had elevated placental cholesterol efflux from maternal to fetal circulation. A similar result was found in a human study reporting that mothers with obesity had significantly lower total plasma cholesterol during the third trimester, but cholesterol was significantly higher in venous cord blood of their newborns in comparison to what was found in normal weight mothers (51). Here, we observed that the level of total cholesterol in plasma of OP mothers was similar to LC mothers, and higher than in OC mothers. Although data in placental tissue is limited, pravastatin treatment has been reported to decrease cholesterol efflux in mouse adipocytes via ATP-binding cassette (ABC) transporters, ABCA1 and ABCG1 (52). Exposure to high cholesterol *in utero* has also been reported to be associated with an elevated risk for cardiovascular and metabolic disorders later in life (53). Therefore, pravastatin administration to mothers with obesity might be protective for fetuses against maternal hypercholesterolemia. Another positive impact of this intervention may be attenuation of the hypothalamic–pituitary–adrenal (HPA) axis dysfunction found in OC infants who showed an enhanced negative glucocorticoid feedback sensitivity by Dex injection. This is an important finding as Dex super-suppressors have been associated with chronic social stress in rhesus macaques (54), and patients with posttraumatic stress disorder (55) and depression (56).

One potential negative impact of gestational pravastatin administration might be on infant kidney function. Both U/P Cr and BCR values (which may be used to assess renal function (43, 44)) were higher in OP compared to LC infants. Considering that plasma creatinine was within the normal range (57) but higher BUN was observed, OP infants could be experiencing an early sign of development of prerenal azotemia (58). However, the impact of gestational pravastatin administration on fetal renal development has not been studied before, and previous publications have found either no toxicity of pravastatin use in patients with kidney problems or improvement in renal function through attenuation of the inflammatory response (59, 60, 61). Further assessment of kidney function is required as the parameters used in this study have limited sensitivity (58), and a high BCR level does not necessarily mean impaired renal function (62).

Pravastatin has been previously reported to alter the diversity, composition, and metabolic profiles of the gut microbiota (63). Although we did not study the gut microbiota, some urinary metabolites derived from microbial activity [e.g., 3-indoxylsulfate (64)] were significantly higher in the OP group compared to the OC group. It could be that pravastatin administration during rhesus macaque pregnancy alters the maternal gut microbiota, which in turn could impact initial colonization of the infant gut microbiota. Indeed, it has been shown that microbiota of the infant are inherited from the mother (65, 66). Whether this has a lasting impact on infant health remains to be determined.

Our statistical analysis on the cytokine/chemokine levels did not demonstrate any significant differences between the intervention groups and the control groups in both mothers and offspring. However, considering that some cytokines/chemokines depicted VIP scores above threshold, certain immune markers may be driving some immune differences between groups. Our VIP analysis depicted different cytokines/chemokines as strong differentiators at each time point during gestation. For example, among mothers, IL-6, IL-1β, and IFN-γ were the strongest differentiators between the OR group compared to the OC group during gestation, whereas IL-8, IL-13, and GM-CSF were the strongest drivers that differentiated the OP and OC groups during gestation. Each of these markers were within the range above the VIP score threshold, indicating the functional importance of these cytokines/chemokines during pregnancy in the obese group. While certain cytokines/chemokines can affect the developing fetus through direct transfer of cytokines to the fetal compartment (67), the cytokines/chemokines indicated as important in both intervention groups compared to the OC group in the mothers have not been shown to cross the placenta. However, similar immune marker differences were observed when comparing infants from the intervention groups to the OC group, such as IL-4, IL-12/23(p40), and MCP-1, suggesting that the maternal immune profile may impact the immune profile of their offspring. The dynamics of the immune milieu in both the mothers and offspring should be further assessed to verify the potential immune-related outcomes of obesity interventions during pregnancy.

The activity of the mTOR pathway was assessed in our study because it is critical in neurodevelopment and its dysregulation has been associated with brain disorders such as autism spectrum disorder and learning disability (16, 68). Our results suggest that while both interventions attenuated the elevated activity of brain mTORC1 in infants born to obese mothers, they appeared to have a minor impact on infant cognition assessed by the VPC test at PD30. However, the HI test conducted at PD110 showed that while the OC group showed aberrant reaction, and gestational calorie restriction resulted in little difference from it, gestational pravastatin administration restored the appropriate affective reaction toward the graded stress and challenges. Although further studies are required to understand the mechanism, this result suggests that there may be a critical role of cholesterol metabolism during pregnancy for brain development in offspring.

In conclusion, although gestational calorie restriction normalized some of the maternal metabolic dysregulation during pregnancy as we hypothesized, it did not result in improvement of infant metabolic and cognitive/social development. While pravastatin administration during pregnancy resulted in a minor improvement in maternal circulating cholesterol levels, as well as function of the HPA axis and emotional development in the offspring, it could have affected the offspring kidney function and development of the gut microbiome. Due to the limited sample size of this study and the fact that only male infants were assessed, these results need to be confirmed in a larger study that includes female offspring. A major strength of this study is the fact that the obese mothers developed obesity as a consequence of over-eating and low activity rather than by ingesting an obesogenic diet, and therefore, the results we obtained are highly relevant to obesity in humans. Our study provides a starting place for further study to determine the long-term impacts of pregnancy interventions for obesity on offspring health. Our study illustrates the importance of metabolic assessment of gestational interventions for obesity, especially with regard to the impact on the offspring.

## Data availability statement

The original contributions presented in the study are publicly available. This data can be found at: https://doi.org/10.5061/dryad.6hdr7sr43.

## Ethics statement

The animal study was reviewed and approved by UC Davis Institutional Animal Care and Use Committee.

## Author contributions

YH wrote the original draft. YH and DK curated, analyzed, and visualized the data. YH and ZZ contributed to methodology and data curation. AT contributed to resources. JC, CH, MB, MG, and JW contributed to methodology, formal analysis, resources, and data curation. CV contributed to funding acquirement, methodology, resources, project administration, and data curation. CW contributed to the conceptualization, funding acquisition, methodology, resources, project administration, and data curation. CS contributed to conceptualization, methodology, resources, funding acquisition, and supervision. All authors reviewed and edited the manuscript.

## Funding

This research was supported by National Institutes of Health award (grant number 1R01HD084203) to CW and CV. CS acknowledges support from Kinsella Endowed Chair in Food, Nutrition and Health. This project was also made possible in part by support from the USDA National Institute of Food and Agriculture Hatch Project (grant numbers 1021411 to CS), the NIH (R24OD010962 to JC), and Intellectual and Developmental Disabilities Research Centers funding (grant number P50HD103526) to JW. The California National Primate Research Center is funded through an NIH grant (grant number P51 OD011107), and the 600 MHz NMR is supported through an NIH grant (grant number 1S10RR011973-01).

## Conflict of interest

The authors declare that the research was conducted in the absence of any commercial or financial relationships that could be construed as a potential conflict of interest.

## Publisher’s note

All claims expressed in this article are solely those of the authors and do not necessarily represent those of their affiliated organizations, or those of the publisher, the editors and the reviewers. Any product that may be evaluated in this article, or claim that may be made by its manufacturer, is not guaranteed or endorsed by the publisher.
