# Supplementary material for: Calorie restriction and pravastatin administration during pregnancy in obese rhesus macaques modulates maternal and infant metabolism and infant brain and behavioral development
Source: Front Nutr. 2023 May 15;10:1146804. doi: 10.3389/fnut.2023.1146804 (PMC10225656; doi:10.3389/fnut.2023.1146804)
Supplement: Supplementary file 1 [file Data_Sheet_1.PDF]

frontiers

Supplementary Material

Calorie restriction and pravastatin administration during pregnancy in obese rhesus macaques modulates maternal and infant metabolism and infant brain and behavioral development

Yu Hasegawa, Danielle HJ. Kim, Zhichao Zhang, Ameer Y. Taha, John P. Capitanio, Casey E. Hogrefe, Melissa D. Bauman, Mari S. Golub, Judy Van de Water, Catherine A. VandeVoort, Cheryl K. Walker, Carolyn M. Slupsky *

*** Correspondence:** Corresponding Author: cslupsky@ucdavis.edu

1 Supplementary Figures and Tables

1.1 Supplementary Figures

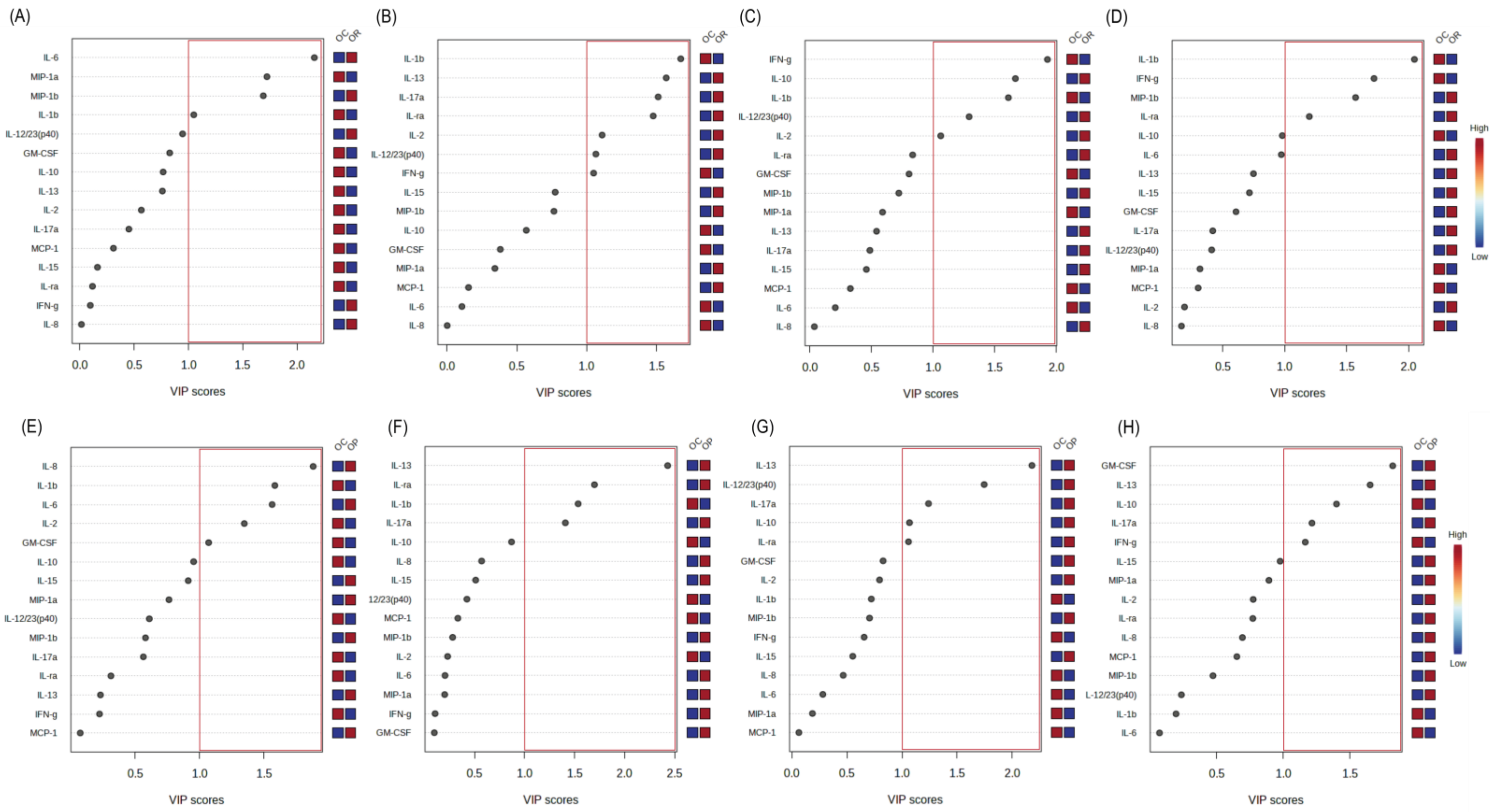

Supplementary Figure 1. Maternal cytokine/chemokine VIP scores for OC group compared to OR or OP group. VIP scores from the comparison between OC and OR groups at (A) GD45, (B) GD90, (C) GD120, (D) GD150; VIP scores from OC and OP groups at (E) GD45, (F) GD90, (G) 120, (H) GD150. Cytokine/chemokines with VIP scores above 1.0 were defined to have meaningful impact on the group

separation with functional importance (indicated by red boxes). The color on the right visualizes the relationships between OC vs OR/OP group; the group with red had higher level of the corresponding marker compared to the group with blue.

Abbreviation: GM-CSF, granulocyte-macrophage colony-stimulating factor; IFN- γ, interferon γ; TNF-α, tumor necrosis factor-α; TGF-α, transforming growth factor-α; MCP-1, monocyte chemoattractant protein-1; MIP-1β, macrophage inflammatory protein-1β; hs-CRP, high-sensitivity C-reactive protein; IL, interleukin.

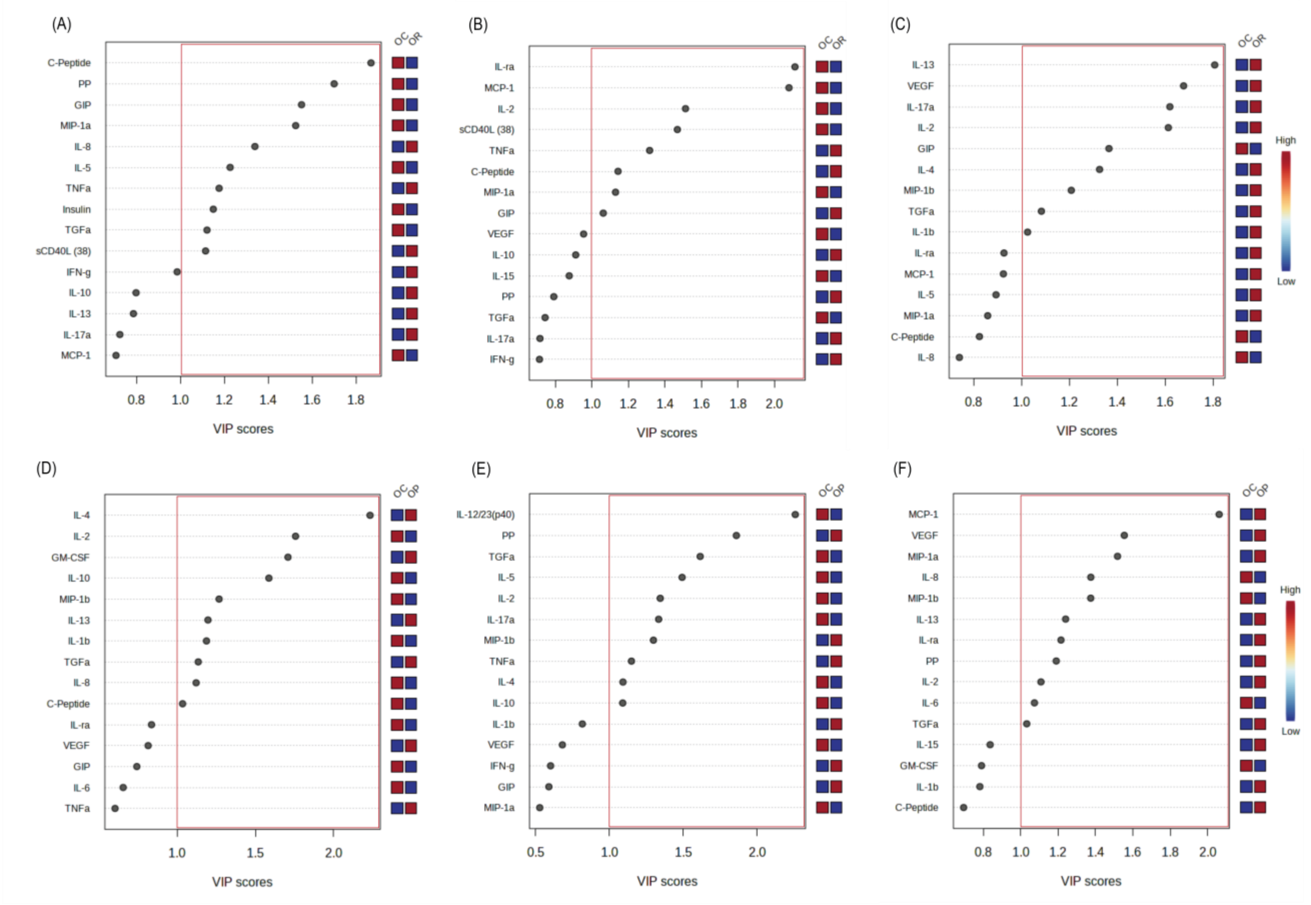

Supplementary Figure 2. Infant cytokine/chemokine VIP scores of OC group compared to OR or OP group. VIP scores from the comparison between OC and OR groups at (A) PD30, (B) PD90, (C) PD180; VIP scores from OC and OP groups at (D) PD30, (E) PD90, (F) PD180. Cytokine/chemokines with VIP scores above 1.0 were defined to have meaningful impact on the group separation with

functional importance (indicated by red boxes). The color on the right visualizes the relationships between OC vs OR/OP group; the group with red had higher level of the corresponding marker compared to the group with blue.

Abbreviation: GM-CSF, granulocyte-macrophage colony-stimulating factor; IFN- γ, interferon γ; TNF-α, tumor necrosis factor-α; TGF-α, transforming growth factor-α; MCP-1, monocyte chemoattractant protein-1; MIP-1β, macrophage inflammatory protein-1β; hs-CRP, high-sensitivity C-reactive protein; IL, interleukin.

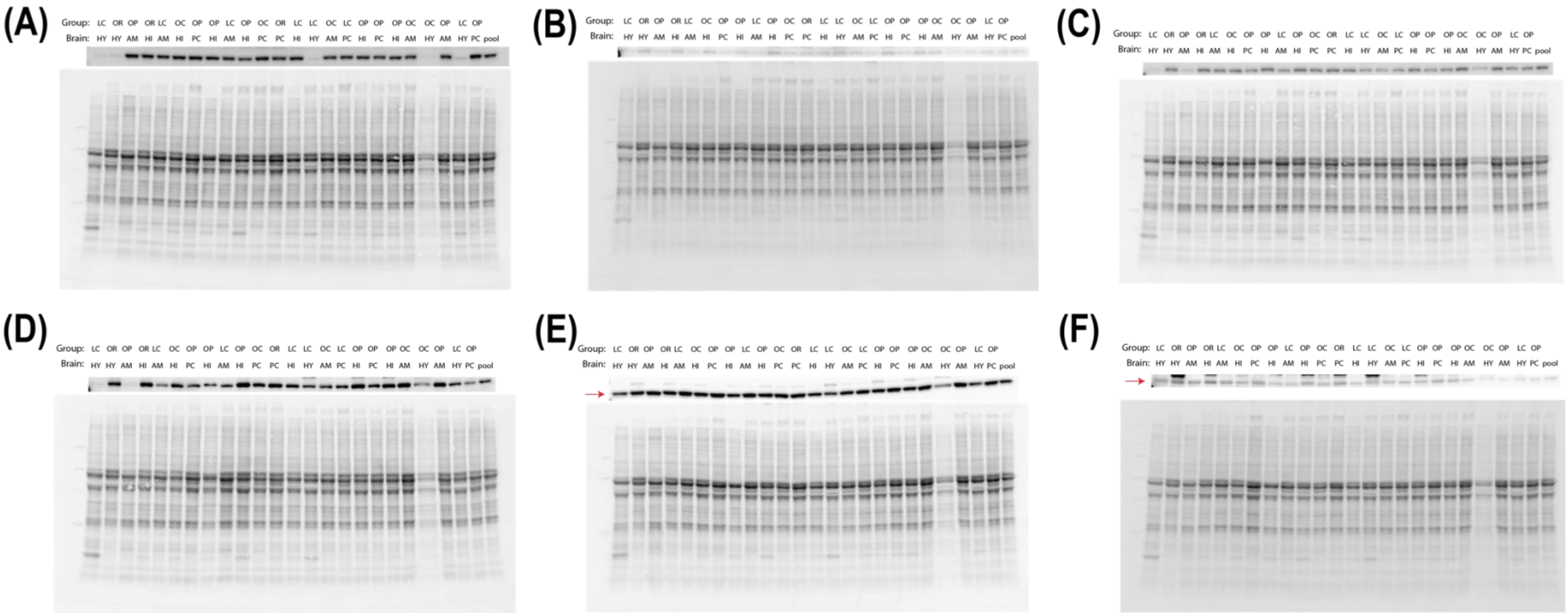

Supplementary Figure 3. The representative Western blotting raw images. Raw images of the bands (above) and the stain-free image showing the total protein (below) are shown for (A) total Akt, (B) p-Akt, (C) total AMPK, (D) p-AMPK, (E) total p70-S6K, and (F) p-p70-S6K. The representative total protein was stained as the endogenous control. The samples from all 4 brain regions were randomized and run with the pooled sample (indicated “pool” in the image) used to normalize between different runs. Samples from different regions of brain were noted with the abbreviation of the brain regions (AM, amygdala; HI, hippocampus; HY, hypothalamus; PC, prefrontal cortex).

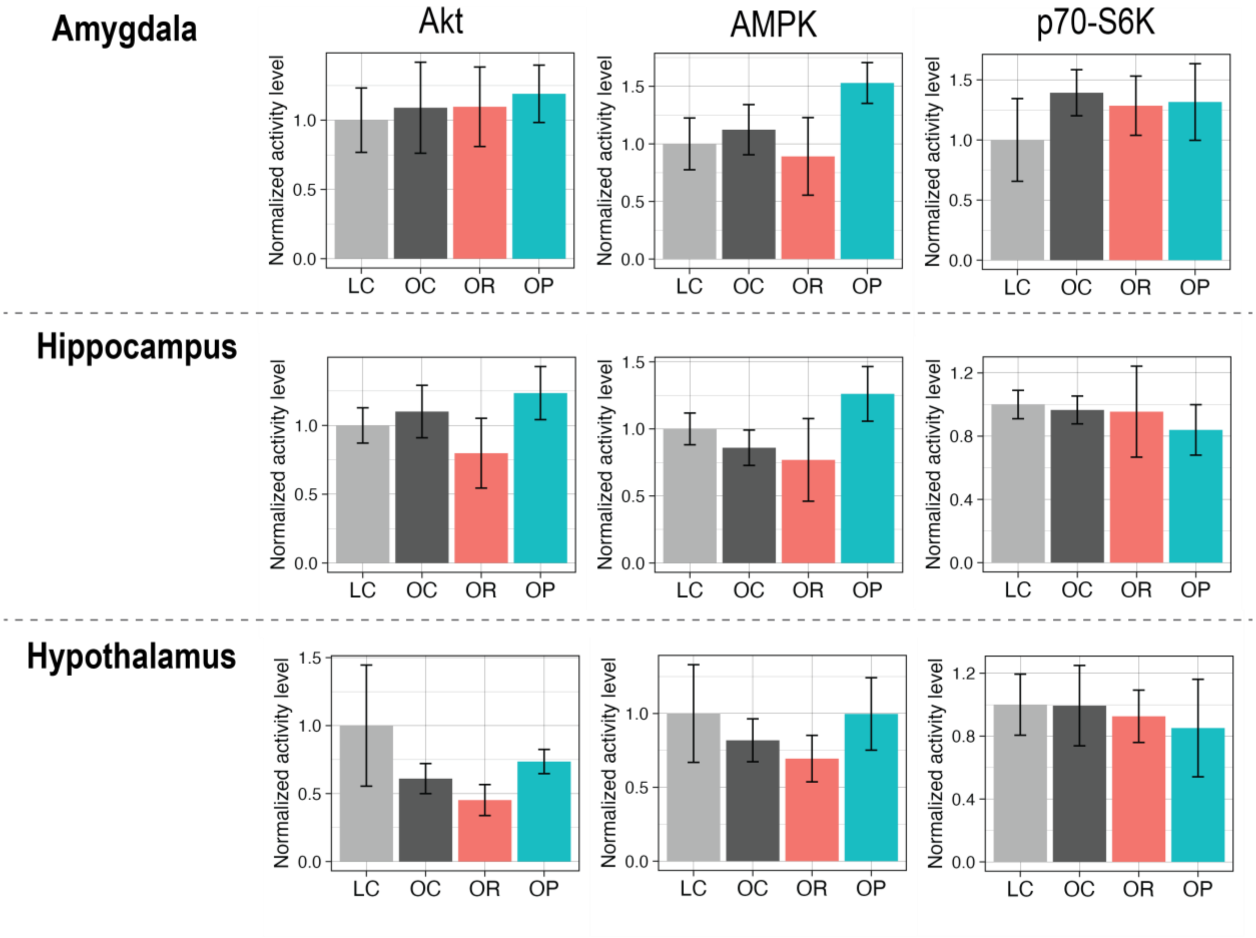

Supplementary Figure 4. Normalized activity of proteins in the mTOR signaling pathway (Akt, AMPK, and p70-S6K) in the amygdala, hippocampus, and hypothalamus. Bar plots are expressed as mean ± standard error. The gray, black, red, and blue colors correspond to the LC, OC, OR, and OP groups, respectively.
